# The risk of open angle glaucoma in young adults with allergic diseases: a Nationwide cohort study

**DOI:** 10.1038/s41598-024-57619-5

**Published:** 2024-05-10

**Authors:** Kyungdo Han, Jin-Hyung Jung, Younhea Jung, Kyoung Ohn, Jung Il Moon

**Affiliations:** 1 Department of Statistics and Actuarial Science, Soongsil University, Seoul, Republic of Korea; 2 Samsung Biomedical Research Institute, Sungkyunkwan University School of Medicine, Suwon, Republic of Korea; 3 Department of Ophthalmology, Yeouido St. Mary’s Hospital, College of Medicine, The Catholic University of Korea, 10, 63-Ro, Yeongdeungpo-Gu, Seoul, 07345 Republic of Korea

**Keywords:** Eye diseases, Respiratory tract diseases, Skin diseases, Diseases

## Abstract

This study investigated the potential associations between allergic diseases (asthma, allergic rhinitis, and atopic dermatitis) and the development of primary open-angle glaucoma. We utilized authorized data from the Korean National Health Information Database (KNHID), which provides comprehensive medical claims data and information from the National Health Screening Program. We compared the baseline characteristics of subjects with and without allergic diseases and calculated the incidence and risk of glaucoma development. Cox proportional hazard regression analysis was used to determine the risk of glaucoma development in subjects with allergic diseases. A total of 171,129 subjects aged 20–39 with or without allergic diseases who underwent a general health examination between 2009 and 2015 were included. Subjects with allergic diseases exhibited a higher incidence of glaucoma compared to the control group. The hazard ratio (HR) of glaucoma onset was 1.49 and 1.39 in subjects with at least one allergic disease before and after adjusting for potential confounding factors, respectively. Among allergic diseases, atopic dermatitis showed the highest risk for glaucoma development (aHR 1.73) after adjusting for confounders. Allergic rhinitis showed an increased risk for incident glaucoma after adjustment (aHR 1.38). Asthma showed the lowest but still increased risk for glaucoma (aHR 1.22). The associations were consistent in all subgroup analyses stratified by sex, smoking, drinking, exercise, diabetes, hypertension, dyslipidemia, or history of steroid. In conclusion, allergic diseases are associated with increased risk of glaucoma development. Among allergic diseases, atopic dermatitis showed the highest risk for glaucoma development followed by allergic rhinitis and asthma.

## Introduction

Allergic diseases are chronic inflammatory disorders triggered by allergens, resulting in immunological responses^1^. They pose a growing health and economic burden, with their prevalence rapidly increasing worldwide^2^. Allergic inflammation, induced by allergen exposure, can lead to various diseases, including asthma, allergic rhinitis (AR), anaphylaxis, urticaria, and atopic dermatitis (AD)^3^. Prolonged or repeated exposure to allergens can cause chronic inflammation not only at the exposed site but also throughout the body, indicating chronic systemic inflammation^4^.

Chronic systemic inflammation plays a critical role in neurodegenerative diseases^5,6^. Allergic inflammation can induce neuroinflammation by activating glial cells in the central and peripheral nervous systems^7^. Consequently, numerous studies have explored the links between allergic diseases and an increased risk of dementia, particularly Alzheimer’s disease, which shares pathophysiological characteristics with glaucoma in respect of cell death mechanisms, cognitive decline, depression, and autism spectrum disorders^8–11^.

Glaucoma, the leading cause of irreversible blindness worldwide, is projected to increase from 76.5 million in 2020 to 111.8 million in 2040^12,13^. Although the prevalence of glaucoma increases with age, the prevalence of primary open-angle glaucoma in those aged 19–29 and 30–39 has been reported to be 1.2% and 2.4%, respectively, in a Nationwide study using the Korea National Health and Nutrition Examination Survey^14^. Identifying risk factors for early detection and treatment is important, especially in these young adults. Glaucoma is a progressive neurodegenerative disease, and neuroinflammation has emerged as an increasingly important risk factor for the development and progression of glaucoma^15,16^. In this regard, chronic systemic inflammation in allergic diseases may be associated with glaucoma pathogenesis. However, only a few studies have examined the associations between allergic diseases and glaucoma, reporting conflicting results^4,7,17^. Therefore, the purpose of this study was to evaluate the potential associations between various allergic diseases, namely asthma, AR, and AD, and the risk of glaucoma development in a large, Nationwide, longitudinal cohort database.

## Method

### Data source

This Nationwide population-based cohort study was approved by the Institutional Review Board of the Yeouido St. Mary’s Hospital, the Catholic University of Korea (SC22ZISE0064). The review board waived the requirement for informed consent because the data were publicly available and anonymous. This research adhered to the tenets of the Declaration of Helsinki. We used the Korean National Health Information Database (KNHID) provided by the Korean National Health Insurance Service (KNHIS). In Korea, all residents are required to be enrolled in the KNHIS. The KNHID includes the following health-related information: (i) demographics including anonymized code for each individual, age, gender, socioeconomic variables, household income, etc., (ii) comprehensive medical data including medical claims data based on diagnostic codes by the International Classification of Diseases 10th revision (ICD-10), admission and ambulatory care, treatment procedures, and prescription records, and (iii) data from the National Health Screening Program (NHSP). The NHSP is conducted by the KNHIS in all enrollees over the age of 20 biannually^18^. It includes basic health examination results such as anthropometric data, visual acuity, pure-tone audiometric testing, blood pressure, basic laboratory exams including fasting glucose and total cholesterol, and a standardized self-questionnaire regarding health-related lifestyle factors (smoking habits, alcohol consumption, and regular physical exercise).

### Study population

We included a total of 587,319 subjects who had undergone a general health examination at least once between 2009 and 2015. The date of the NHSP was regarded as the index date. Only those aged 20–39 were included in the study (n = 179,501). Those with missing data (n = 4552) and those with previously diagnosed glaucoma (n = 3820) were excluded. Previously diagnosed glaucoma was defined as diagnosis of glaucoma between January 1, 2002 and index date. In total, 171,129 subjects were included in the study and were followed using their medical records until December 31, 2018.

Allergic diseases were defined as at least 3 visits to the hospital with ICD-10 diagnostic code for AD (L20), AR (J301-304), or asthma (J45-46) as previously defined^19^ within one year prior to the index date. History of steroid was defined as any type of topical, inhaled, or oral steroid within 1 year of index date. Primary end point was development of primary open angle glaucoma, which was defined as at least 3 visits for glaucoma (H401) as previously defined^20–22^. The first diagnosis date was regarded as the occurrence date.

Health examinations were performed in hospitals certified by the KNHIS. If a subject underwent more than one health examination, only the first health examination was included for analysis. Anthropometric measurements were taken with the subjects wearing light clothing. Body mass index was calculated as the weight (kg) divided by the square of the height (m). Blood samples for serum glucose and lipid profiles including total cholesterol, high-density lipoprotein (HDL) cholesterol, and low-density lipoprotein (LDL) cholesterol were collected after an overnight fasting. Blood pressure was measured in a sitting position after a 5-min rest.

Comorbidities including diabetes (E11–E14), hypertension (I10, I11, I12, I13, and I15), and hypercholesterolemia (E78) were also defined based on ICD-10 codes and prescription history^19,22^. Smoking status was classified into non-smoker, ex-smoker, or current smoker, and alcohol consumption was classified into non-drinker (no alcoholic drinks within the past year), mild drinker (< 30 g of alcohol per day), or heavy drinker (≥ 30 g of alcohol per day). Subjects’ socioeconomic status was dichotomized into upper 80% and lower 20% based on household income, and area of residency was classified into urban or rural.

### Statistical analysis

The baseline characteristics of the study subjects were compared using the student’s t-test for continuous variables and *X*^2^ test for categorical variables. The incidence rate of glaucoma was calculated by dividing the number of events by 1,000 person-years. Cox proportional hazard regression analysis was used to calculate the risk of glaucoma development according to the state of allergic diseases. Hazard ratio (HR) and 95% confidence interval (CI) was calculated before and after adjusting for potential confounding factors. Fully adjusted model included age, sex, income, hypertension, dyslipidemia, smoking status, alcohol consumption, regular exercise, body mass index (BMI), and history of steroid. In addition, we also calculated the risk of glaucoma development with at least one year lag after the index date to establish a temporal relationship. Subgroup analysis was performed after stratification according to sex, smoking status, alcohol consumption, regular exercise, comorbidities including diabetes, hypertension, dyslipidemia, and use of steroid. Interaction terms of allergic diseases with each stratification category were added to the Cox model to test the significance of the subgroup effects, and p values for interaction was calculated. Kaplan–Meier curve for incidence probability of glaucoma was generated.

## Results

### Baseline characteristics of the study population

A total of 171,129 subjects were included in the study. Table 1 shows the baseline characteristics of the study subjects. Subjects with at least one allergic disease (n = 23,758) were more likely female, in the lowest income quintile, non-smoker, non-drinker, older, lower BMI, lower glucose level, lower systolic and diastolic blood pressure, and better lipid profiles (lower total cholesterol, higher HDL, and lower LDL). Subjects with allergic disease were less likely to have comorbidities including hypertension and dyslipidemia, but not diabetes. Of note, subjects with at least one allergic disease were more likely to have received steroid treatment (topical, inhaled, or oral steroid) within 1 year of index date.

**Table 1:** Baseline characteristics of study subjects.

n	Atopic dermatitis, allergic rhinitis, or asthma			Atopic dermatitis		
	No	Yes	P-value	No	Yes	P-value
	147,371	23,758		170,319	810	
Sex, male	88,562 (60.1)	9570 (40.3)	< 0.01	97,768 (57.4)	364 (44.9)	< 0.01
Income, low 20%	23,430 (15.9)	4821 (20.3)	< 0.01	28,062 (16.5)	189 (23.3)	< 0.01
Smoking			< 0.01			< 0.01
Non	79,484 (53.9)	15,994 (67.3)		94,944 (55.7)	534 (65.9)	
Ex	14,839 (10.1)	2441 (10.3)		17,197 (10.1)	83 (10.3)	
Current	53,048 (36.0)	5323 (22.4)		58,178 (34.2)	193 (23.8)	
Alcohol			< 0.01			< 0.01
Non	51,937 (35.2)	10,587 (44.6)		62,164 (36.5)	360 (44.4)	
Mild	81,347 (55.2)	11,707 (49.3)		92,655 (54.4)	399 (49.3)	
Heavy	14,087 (9.6)	1464 (6.2)		15,500 (9.1)	51 (6.3)	
Regular exercise	20,140 (13.7)	3153 (13.3)	0.10	23,177 (13.6)	116 (14.3)	0.55
Diabetes	2811 (1.9)	439 (1.9)	0.53	3239 (1.9)	11 (1.4)	0.26
Hypertension	10,695 (7.3)	1518 (6.4)	< 0.01	12,169 (7.1)	44 (5.4)	0.06
Dyslipidemia	10,107 (6.9)	1525 (6.4)	0.01	11,590 (6.8)	42 (5.2)	0.07
Steroid use	19,777 (13.4)	9179 (38.6)	< 0.01	28,489 (16.7)	467 (57.7)	< 0.01
Age, year	30.28 ± 5.12	31.00 ± 5.21	< 0.01	30.39 ± 5.14	28.79 ± 5.1	< 0.01
Body mass index, kg/m^2^	23.10 ± 3.7	22.78 ± 3.75	< 0.01	23.06 ± 3.71	22.67 ± 3.86	< 0.01
Blood glucose, mg/dL	91.10 ± 17.46	90.46 ± 15.98	< 0.01	91.02 ± 17.28	89.06 ± 15.35	< 0.01
Systolic blood pressure, mmHg	117.80 ± 13.18	115.48 ± 12.89	< 0.01	117.48 ± 13.17	115.56 ± 12.72	< 0.01
Diastolic blood pressure, mmHg	73.74 ± 9.45	72.36 ± 9.4	< 0.01	73.56 ± 9.45	72.35 ± 9.18	< 0.01
Total cholesterol, mg/dL	184.25 ± 33.78	182.62 ± 32.9	< 0.01	184.04 ± 33.68	181.45 ± 31.58	< 0.01
High density lipoprotein, mg/dL	57.71 ± 20.19	58.69 ± 21.22	< 0.01	57.84 ± 20.37	58.50 ± 13.81	< 0.01
Low density lipoprotein, mg/dL	104.25 ± 33.14	103.44 ± 32.76	< 0.01	104.15 ± 33.11	102.40 ± 27.99	< 0.01

n	Allergic rhinitis			Asthma		
	No	Yes	P-value	No	Yes	P-value
	148,508	22,621		168,961	2168	
Sex, male	89,111 (60.0)	9021 (39.9)	< 0.01	97,240 (57.6)	892 (41.1)	< 0.01
Income, low 20%	23,663 (15.9)	4588 (20.3)	< 0.01	27,776 (16.4)	475 (21.9)	< 0.01
Smoke			< 0.01			< 0.01
Non	80,193 (54.0)	15,285 (67.6)		94,035 (55.7)	1443 (66.6)	
Ex	14,960 (10.1)	2320 (10.3)		17,036 (10.)	244 (11.3)	
Current	53,355 (35.9)	5016 (22.2)		57,890 (34.3)	481 (22.2)	
Drink			< 0.01			< 0.01
Non	52,452 (35.3)	10,072 (44.5)		61,491 (36.4)	1033 (47.7)	
Mild	81,891 (55.1)	11,163 (49.4)		92,046 (54.5)	1008 (46.5)	
Heavy	14,165 (9.5)	1386 (6.1)		15,424 (9.1)	127 (5.9)	
Regular exercise	20,304 (13.7)	2989 (13.2)	0.06	23,018 (13.6)	275 (12.7)	0.21
Diabetes	2843 (1.9)	407 (1.8)	0.24	3192 (1.9)	58 (2.7)	0.01
Hypertension	10,774 (7.3)	1439 (6.4)	< 0.01	12,049 (7.1)	164 (7.6)	0.44
Dyslipidemia	10,169 (6.9)	1463 (6.5)	0.03	11,484 (6.8)	148 (6.8)	0.96
Steroid use	20,334 (13.7)	8622 (38.1)	< 0.01	27,863 (16.5)	1093 (50.4)	< 0.01
Age, year	30.28 ± 5.12	31.05 ± 5.21	< 0.01	30.37 ± 5.14	31.73 ± 5.02	< 0.01
Body mass index, kg/m^2^	23.10 ± 3.7	22.76 ± 3.74	< 0.01	23.05 ± 3.71	23.14 ± 4.01	< 0.01
Blood glucose, mg/dL	91.09 ± 17.47	90.44 ± 15.87	< 0.01	91.01 ± 17.26	90.91 ± 17.92	< 0.01
Systolic blood pressure, mmHg	117.79 ± 13.18	115.44 ± 12.90	< 0.01	117.49 ± 13.16	116.13 ± 13.05	< 0.01
Diastolic blood pressure, mmHg	73.73 ± 9.45	72.34 ± 9.40	< 0.01	73.56 ± 9.45	72.99 ± 9.38	< 0.01
Total cholesterol, mg/dL	184.24 ± 33.77	182.60 ± 32.92	< 0.01	184.02 ± 33.66	184.49 ± 34.01	< 0.01
High density lipoprotein, mg/dL	57.71 ± 20.20	58.72 ± 21.24	< 0.01	57.85 ± 20.34	57.86 ± 20.31	< 0.01
Low density lipoprotein, mg/dL	104.25 ± 33.11	103.44 ± 32.94	< 0.01	104.12 ± 33.05	105.58 ± 36.33	< 0.01

*POAG* primary open-angle glaucoma.

### Incidence and risk of glaucoma among patients with allergic diseases

Table 2 shows the incidence and risk of glaucoma onset according to allergic diseases. The incidence of glaucoma was 4.50 and 7.08 per 1000 person-years in the control group and allergic disease group, respectively. Among allergic diseases, the incidence of glaucoma was 4.83 and 9.35 per 1000 person-years in the control group and AD group, respectively; 4.53 and 7.07 per 1000 person-years in the control group and AR group, respectively; and 4.82 and 7.07 per 1000 person-years in the control group and asthma group, respectively. The risk of glaucoma onset was 1.49 (95% CI 1.40–1.58) in subjects with at least one allergic disease before adjusting for potential confounding factors. After adjustment, the adjusted HR was 1.39 (95% CI 1.30–1.48). Among allergic diseases, AD showed the highest risk for glaucoma development before (HR 1.97, 95% CI 1.52–2.56) and after (aHR 1.73, 95% CI 1.34–2.24) adjustment. Allergic rhinitis showed increased risk for incident glaucoma before (HR 1.47, 95% CI 1.39–1.57) and after (aHR 1.38, 95% CI 1.29–1.47) adjusting for confounders. Among allergic diseases, asthma showed the lowest, but still increased risk for glaucoma before (HR 1.37, 95% CI 1.14–1.63) and after (aHR 1.22, 95% CI 1.02–1.46) adjustment. In addition, Supplementary Table 1 shows the risk of glaucoma including only those with at least one year lag between the index date and glaucoma, which shows similar results.

**Table 2:** Risk of primary open-angle glaucoma development in allergic diseases.

	N	POAG	Duration	Incidence rate (per 1000)	Model 1	Model 2	Model 3
Atopic dermatitis, allergic rhinitis, or asthma							
No	147,371	5495	1,219,786.20	4.50	1 (ref.)	1 (ref.)	1 (ref.)
Yes	23,758	1326	187,262.32	7.08	1.49 (1.40, 1.58)	1.48 (1.39, 1.57)	1.39 (1.30, 1.48)
Atopic dermatitis							
No	170,319	6763	1,400,843.58	4.83	1 (ref.)	1 (ref.)	1 (ref.)
Yes	810	58	6204.95	9.35	1.97 (1.52, 2.56)	1.96 (1.51, 2.54)	1.73 (1.34, 2.24)
Allergic rhinitis							
No	148,508	5562	1,228,976.3	4.53	1 (ref.)	1 (ref.)	1 (ref.)
Yes	22,621	1259	178,072.23	7.07	1.47 (1.39, 1.57)	1.46 (1.37, 1.56)	1.38 (1.29, 1.47)
Asthma							
No	168,961	6700	1,389,936.19	4.82	1 (ref.)	1 (ref.)	1 (ref.)
Yes	2168	121	17,112.33	7.07	1.37 (1.14, 1.63)	1.35 (1.13, 1.62)	1.22 (1.02, 1.46)

Model 1: Age, Sex.

Model 2: Age, Sex, Income, hypertension, dyslipidemia, smoking, drinking, exercise, and body mass index.

Model 3: Age, Sex, Income, hypertension, dyslipidemia, smoking, drinking, exercise, body mass index, and steroid use.

*POAG* primary open-angle glaucoma.

**Figure 1:**
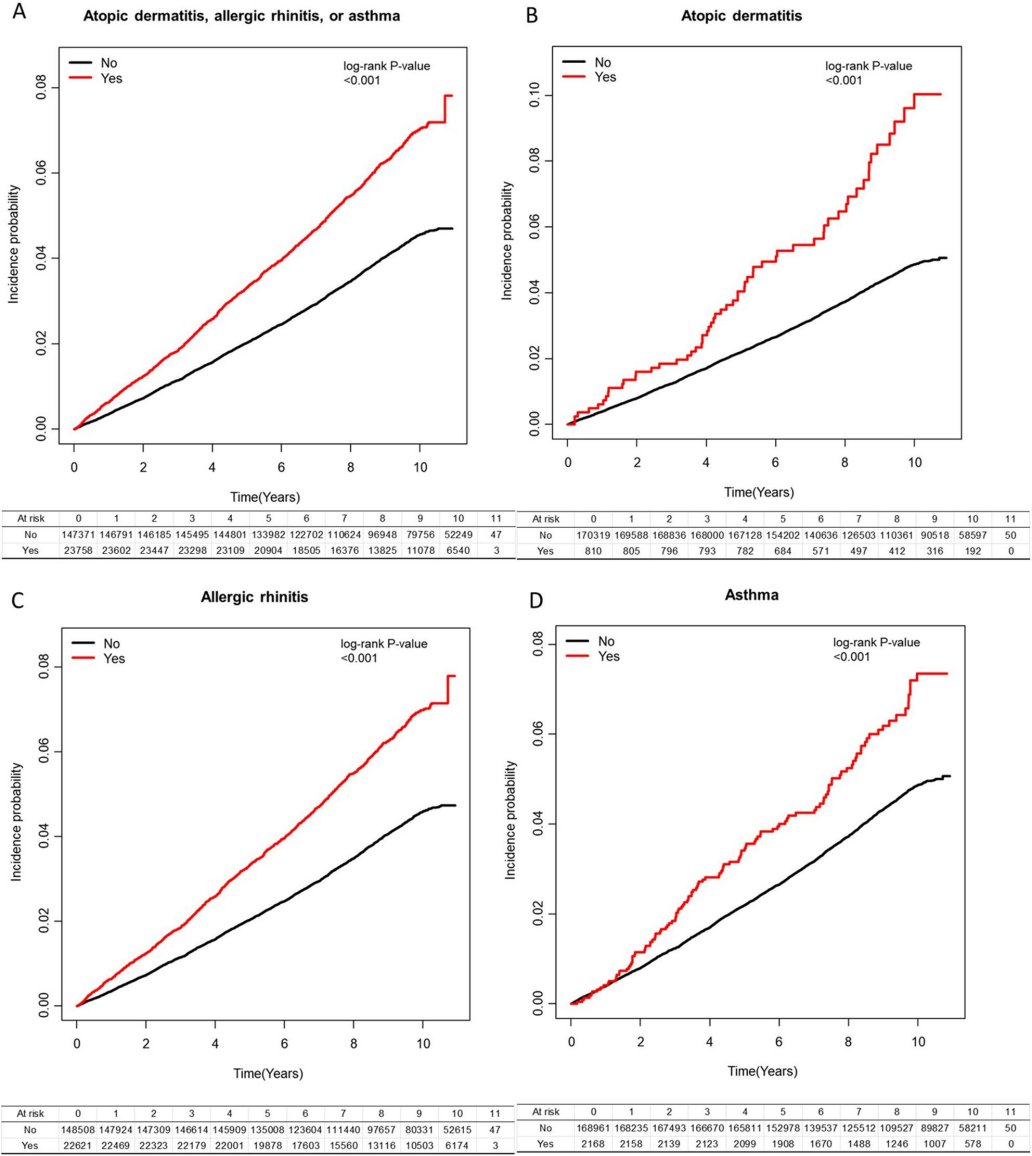
Cumulative glaucoma incidence according to allergic diseases.

In subgroup analyses, adjusted HRs of study outcomes were compared in each subgroup stratified by sex, smoking, drinking, exercise, diabetes, hypertension, dyslipidemia, or history of steroid (Table 3). Overall, the association between allergic diseases and glaucoma onset was consistent in all subgroup analyses. In subjects with AD, those with dyslipidemia showed significantly greater risk of incident glaucoma (P for interaction = 0.01) compared to those without. In subjects with AR, those who are current smokers showed significantly greater risk of glaucoma development (P for interaction = 0.03) than those who are non-current smokers. In addition, those with history of steroid use showed significantly less prominent association with glaucoma onset compared to those without (P for interaction = 0.03). In subjects with asthma, the association between asthma and glaucoma was consistent in all subgroups. Figure 1 shows the cumulative glaucoma incidence according to allergic diseases.

**Table 3:** Risk of primary open-angle glaucoma development in allergic diseases stratified by sex, smoking, drinking, exercise, diabetes, hypertension, dyslipidemia, and steroid use.

	Atopic dermatitis, allergic rhinitis, or asthma						Atopic dermatitis					
	N	POAG	Duration	IR (per 1000)	Adjusted HR	P for interaction	N	POAG	Duration	IR (per 1000)	Adjusted HR	P for interaction
Male												
No	88,562	3087	742,779.67	4.16	1 (ref.)	0.79	97,768	3565	817,758.73	4.36	1 (ref.)	0.09
Yes	9570	508	77,816.10	6.53	1.40 (1.27, 1.54)		364	30	2837.04	10.57	2.21 (1.54, 3.17)	
Female												
No	58,809	2408	477,006.53	5.05	1 (ref.)		72,551	3198	583,084.84	5.48	1 (ref.)	
Yes	14,188	818	109,446.22	7.47	1.38 (1.27, 1.50)		446	28	3367.91	8.31	1.41 (0.97, 2.04)	
Smoke, non												
No	79,484	3212	648,504.99	4.95	1 (ref.)	0.07	94,944	4091	769,349.46	5.32	1 (ref.)	0.34
Yes	15,994	914	124,862.95	7.32	1.35 (1.25, 1.46)		534	35	4018.48	8.71	1.51 (1.08, 2.11)	
Smoke, ex												
No	14,839	616	125,741.75	4.90	1 (ref.)		17,197	745	145,020.78	5.14	1 (ref.)	
Yes	2441	137	19,890.92	6.89	1.28 (1.06, 1.54)		83	8	611.89	13.07	2.37 (1.18, 4.77)	
Smoke, current												
No	53,048	1667	445,539.46	3.74	1 (ref.)		58,178	1927	486,473.34	3.96	1 (ref.)	
Yes	5323	275	42,508.45	6.47	1.58 (1.39, 1.80)		193	15	1574.58	9.53	2.15 (1.29, 3.58)	
Drink, non												
No	51,937	2067	431,558.58	4.79	1 (ref.)	0.52	62,164	2681	512,551.33	5.23	1 (ref.)	0.39
Yes	10,587	641	83,753.52	7.65	1.44 (1.32, 1.58)		360	27	2760.77	9.78	1.70 (1.16, 2.49)	
Drink, mild												
No	81,347	2970	671,566.20	4.42	1 (ref.)		92,655	3560	760,447.63	4.68	1 (ref.)	
Yes	11,707	616	91,949.85	6.70	1.34 (1.23, 1.47)		399	26	3068.42	8.47	1.62 (1.10, 2.38)	
Drink, heavy												
No	14,087	458	116,661.42	3.93	1 (ref.)		15,500	522	127,844.61	4.08	1 (ref.)	
Yes	1464	69	11,558.95	5.97	1.374 (1.07, 1.77)		51	5	375.76	13.31	3.14 (1.30, 7.58)	
Regular exercise, no												
No	127,231	4750	1,054,649.75	4.50	1 (ref.)	0.39	147,142	5862	1,211,805.41	4.84	1 (ref.)	0.93
Yes	20,605	1162	162,453.01	7.15	1.40 (1.31, 1.50)		694	50	5297.35	9.44	1.74 (1.32, 2.30)	
Regular exercise, yes												
No	20,140	745	165,136.45	4.51	1 (ref.)		23,177	901	189,038.17	4.77	1 (ref.)	
Yes	3153	164	24,809.31	6.61	1.30 (1.09, 1.54)		116	8	907.59	8.81	1.68 (0.84, 3.38)	
Diabetes (−)												
No	144,560	5314	1,196,385.75	4.44	1 (ref.)	0.17	167,080	6549	1,374,008.75	4.77	1 (ref.)	0.87
Yes	23,319	1293	183,739.87	7.04	1.40 (1.31, 1.49)		799	58	6116.88	9.48	1.77 (1.36, 2.29)	
Diabetes (+)												
No	2811	181	23,400.45	7.73	1 (ref.)		3239	214	26,834.83	7.97	1 (ref.)	
Yes	439	33	3522.45	9.37	1.07 (0.74, 1.55)		11	0	88.07	0.00	–	
Hypertension (−)												
No	136,676	5042	1,129,124.72	4.47	1 (ref.)	0.28	158,150	6226	1,298,401.04	4.80	1 (ref.)	0.18
Yes	22,240	1241	175,129.84	7.09	1.40 (1.31, 1.50)		766	57	5853.51	9.74	1.82 (1.40, 2.36)	
Hypertension (+)												
No	10,695	453	90,661.48	5.00	1 (ref.)		12,169	537	102,442.54	5.24	1 (ref.)	
Yes	1518	85	12,132.49	7.01	1.23 (0.97, 1.55)		44	1	351.44	2.85	0.47 (0.07, 3.34)	
Dyslipidemia (−)												
No	137,264	5052	1,135,392.21	4.45	1 (ref.)	0.80	158,729	6231	1,304,579.87	4.78	1 (ref.)	0.01
Yes	22,233	1229	175,080.67	7.02	1.39 (1.30, 1.49)		768	50	5893.02	8.48	1.58 (1.20, 2.09)	
Dyslipidemia (+)												
No	10,107	443	84,393.99	5.25	1 (ref.)		11,590	532	96,263.71	5.53	1 (ref.)	
Yes	1525	97	12,181.65	7.96	1.35 (1.08, 1.69)		42	8	311.93	25.65	4.09 (2.04, 8.23)	
Steroid (−)												
No	127,594	4546	1,057,068.18	4.30	1 (ref.)	0.06	141,830	5290	1,169,621.65	4.52	1 (ref.)	0.82
Yes	14,579	765	115,161.71	6.64	1.45 (1.35, 1.57)		343	21	2608.24	8.05	1.80 (1.17, 2.77)	
Steroid (+)												
No	19,777	949	162,718.02	5.83	1 (ref.)		28,489	1473	231,221.93	6.37	1 (ref.)	
Yes	9179	561	72,100.62	7.78	1.28 (1.15, 1.42)		467	37	3596.71	10.29	1.69 (1.22, 2.35)	

	Allergic rhinitis						Asthma					
	N	POAG	Duration	IR (per 1,000)	Adjusted HR	P for interaction	N	POAG	Duration	IR (per 1,000)	Adjusted HR	P for interaction
Male												
No	89,111	3124	747,291.44	4.18	1 (ref.)	0.86	97,240	3547	813,276.61	4.36	1 (ref.)	0.79
Yes	9021	471	73,304.33	6.43	1.37 (1.24, 1.51)		892	48	7319.16	6.56	1.26 (0.95, 1.68)	
Female												
No	59,397	2438	481,684.85	5.06	1 (ref.)		71,721	3153	576,659.59	5.47	1 (ref.)	
Yes	13,600	788	104,767.90	7.52	1.38 (1.28, 1.50)		1276	73	9793.16	7.45	1.20 (0.95, 1.51)	
Smoke, non												
No	80,193	3255	654,158.55	4.98	1 (ref.)	0.03	94,035	4044	762,058.04	5.31	1 (ref.)	0.68
Yes	15,285	871	119,209.39	7.31	1.34 (1.24, 1.45)		1443	82	11,309.90	7.25	1.17 (0.94, 1.46)	
Smoke, ex												
No	14,960	627	126,699.47	4.95	1 (ref.)		17,036	739	143,671.40	5.14	1 (ref.)	
Yes	2320	126	18,933.20	6.65	1.22 (1.01, 1.48)		244	14	1961.27	7.14	1.19 (0.70, 2.01)	
Smoke, current												
No	53,355	1680	448,118.28	3.75	1 (ref.)		57,890	1917	484,206.76	3.96	1 (ref.)	
Yes	5016	262	39,929.64	6.56	1.60 (1.40, 1.82)		481	25	3841.16	6.51	1.43 (0.97, 2.13)	
Drink, non												
No	52,452	2104	435,692.92	4.83	1 (ref.)	0.68	61,491	2642	507,167.96	5.21	1 (ref.)	0.58
Yes	10,072	604	79,619.19	7.59	1.42 (1.29, 1.55)		1033	66	8144.14	8.10	1.33 (1.04, 1.70)	
Drink, mild												
No	81,891	2998	676,013.13	4.43	1 (ref.)		92,046	3537	755,533.15	4.68	1 (ref.)	
Yes	11,163	588	87,502.92	6.72	1.34 (1.22, 1.47)		1008	49	7982.90	6.14	1.10 (0.83, 1.45)	
Drink, heavy												
No	14,165	460	117,270.25	3.92	1 (ref.)		15,424	521	127,235.08	4.09	1 (ref.)	
Yes	1386	67	10,950.12	6.12	1.40 (1.09, 1.81)		127	6	985.29	6.09	1.26 (0.56, 2.82)	
Regular exercise, no												
No	128,204	4805	1,062,504.76	4.52	1 (ref.)	0.27	145,943	5806	1,202,165.22	4.83	1 (ref.)	1.00
Yes	19,632	1107	154,598.00	7.16	1.40 (1.30, 1.49)		1893	106	14,937.55	7.10	1.22 (1.01, 1.48)	
Regular exercise, yes												
No	20,304	757	166,471.53	4.55	1 (ref.)		23,018	894	187,770.98	4.76	1 (ref.)	
Yes	2989	152	23,474.23	6.48	1.26 (1.06, 1.50)		275	15	2174.78	6.90	1.22 (0.73, 2.03)	
Diabetes (−)												
No	145,665	5380	1,205,324.07	4.46	1 (ref.)	0.28	165,769	6491	1,363,464.78	4.76	1 (ref.)	0.94
Yes	22,214	1227	174,801.55	7.02	1.39 (1.30, 1.48)		2110	116	16,660.84	6.96	1.22 (1.01, 1.47)	
Diabetes (+)												
No	2843	182	23,652.22	7.69	1 (ref.)		3192	209	26,471.41	7.90	1 (ref.)	
Yes	407	32	3270.68	9.78	1.12 (0.77, 1.63)		58	5	451.49	11.07	1.18 (0.49, 2.85)	
Hypertension (−)												
No	137,734	5107	1,137,679.76	4.49	1 (ref.)	0.46	156,912	6170	1,288,440.82	4.79	1 (ref.)	0.48
Yes	21,182	1176	166,574.79	7.06	1.39 (1.30, 1.48)		2004	113	15,813.73	7.15	1.25 (1.03, 1.50)	
Hypertension (+)												
No	10,774	455	91,296.54	4.98	1 (ref.)		12,049	530	101,495.38	5.22	1 (ref.)	
Yes	1439	83	11,497.44	7.22	1.27 (1.00, 1.60)		164	8	1298.59	6.16	0.96 (0.48, 1.94)	
Dyslipidemia (−)												
No	138,339	5113	1,144,077.76	4.47	1 (ref.)	0.65	157,477	6166	1,294,565.71	4.76	1 (ref.)	0.21
Yes	21,158	1168	166,395.13	7.02	1.38 (1.30, 1.48)		2020	115	15,907.18	7.23	1.26 (1.05, 1.52)	
Dyslipidemia (+)												
No	10,169	449	84,898.54	5.29	1 (ref.)		11,484	534	95,370.48	5.60	1 (ref.)	
Yes	1463	91	11,677.10	7.79	1.31 (1.05, 1.64)		148	6	1205.15	4.98	0.75 (0.33, 1.67)	
Steroid (−)												
No	128,174	4576	1,061,791.96	4.31	1 (ref.)	0.03	141,098	5254	1,163,731.61	4.51	1 (ref.)	0.22
Yes	13,999	735	110,437.93	6.66	1.45 (1.34, 1.57)		1075	57	8498.28	6.71	1.38 (1.07, 1.80)	
Steroid (+)												
No	20,334	986	167,184.34	5.90	1 (ref.)		27,863	1446	226,204.59	6.39	1 (ref.)	
Yes	8622	524	67,634.30	7.75	1.26 (1.13, 1.40)		1093	64	8614.05	7.43	1.10 (0.86, 1.42)

## Discussion

In this Nationwide longitudinal cohort study including young adults, we found significantly increased risk of glaucoma development in allergic diseases in general and in each of the three most common allergic diseases per se. Among allergic diseases, AD showed the greatest risk of subsequent glaucoma onset, followed by AR and asthma. Of note, the association was significant before and after adjusting for potential confounding factors including history of steroid use. The association between allergic diseases and subsequent glaucoma onset was robustly significant in all subgroups stratified by gender, lifestyle factors including smoking, drinking, and exercise, and systemic diseases including diabetes, hypertension, dyslipidemia, and history of steroid use.

There are few previous studies investigating the association between allergic diseases and glaucoma, with conflicting results. While glaucoma was not associated with allergic diseases in a Nationwide cross-sectional study using the Korean National Health and Nutrition Examination Survey^4^, it was associated with increased risk of AR using a population-based data in Taiwan^17^. In this study, Chung et al.^17^ hypothesized that the relationship between AR and glaucoma was mediated by autonomic dysfunction, as patients with AR were shown to have poor sympathetic modulation^23^, and nitric oxide, as high levels of nitric oxide were reported in patients with allergies^24^. In another study, Tseng et al.^7^ reported that glaucoma was associated with higher odds of sensitization to cockroach and cat allergens using the National Health and Nutrition Examination Survey, indicating that chronic exposure to certain allergens may play a role in the development of glaucoma. However, these studies were cross-sectional, limited to a particular allergic disease, or limited to self-reported allergic diseases. We found allergic diseases, namely AD, AR, and asthma, were in general and independently associated with increased risk of glaucoma development in a large Nationwide longitudinal cohort.

Although the exact pathophysiological mechanisms between allergic diseases and glaucoma remain unknown, there are some plausible etiologies. Chronic systemic inflammation is known to be associated with neurodegeneration^12^. Persistent or repetitive exposure to allergens in allergic diseases lead to chronic inflammation not only at the site of exposure, but even at other sites throughout the body^3^. Chronic systemic inflammation has been shown to stimulate glial cells to express proinflammatory cytokines including TNFα, IL-1, IL-4, IL-6, and IL-10^8^. Elevated levels of these proinflammatory cytokines near the optic nerve head could potentially lead to neuroinflammation and neurodegeneration of the optic nerve. In line with this potential mechanism, the risk of incident dementia and Alzheimer's disease was increased in patients with asthma, AR, and AD, with dose–effect relationship with the severity of allergic diseases in a previous study^8^. The authors also explained the potential links between allergic diseases and dementia were chronic systemic inflammation and immune alterations affecting the brain. In a previous study, patients with glaucoma showed higher serum levels of IL-4 and IL-6 and those with severe optic neuropathy showed even higher levels, also suggesting the role of abnormal immune environment in the glaucoma pathogenesis^25^.

Oxidative stress in allergic diseases may also act as a link. Reactive oxygen species and reactive nitrogen species are increased in allergic diseases^26^. In asthma, not only airway, but also circulating inflammatory cells can secrete superoxide and contribute to elevated oxidative stress in asthma^26,27^. In addition, peripheral blood monocytes are primed to generate toxic oxygen metabolites in patients with severe AD^28^. Oxidative stress plays an important role in the pathogenesis of glaucoma causing trabecular meshwork degeneration and death of retinal ganglion cells^29,30^. In addition, allergic diseases have been linked with endothelial dysfunction, and therefore has been associated with systemic vascular diseases including diabetes, hypertension, and cardiovascular diseases^8^. Systemic vascular endothelial dysfunction has been associated with glaucoma, especially normal tension glaucoma which is the most prevalent type of primary open angle glaucoma in Korea^31,32^.

We found that among allergic diseases, AD showed the greatest risk of subsequent development of glaucoma. Repetitive and continuous eye rubbing can elevate intraocular pressure and has been associated with glaucomatous optic neuropathy^33,34^ Pecora and associates reported a case of a patient with progressive “normal tension glaucoma” which seemed to have been caused by recurrent eye rubbing. Progressive deterioration stabilized after the patient stopped the habit^34^.

In addition, steroid can cause elevation in IOP resulting in steroid-induced glaucoma, which may confound the association between allergic diseases and glaucoma^35^. Although steroid induced glaucoma (H406) may not likely be included in the analyses since we included only those with primary open angle glaucoma (H401), however, we attempted to further account for this by controlling for steroid in our analyses, which revealed consistent results. Furthermore, AR subjects with history of steroid use showed less prominent association with incident glaucoma compared to those without. Although exact mechanism remains unknown, potential mechanism may involve reduced inflammation by steroid. A previous study also reported decreased incidence of glaucoma in children with asthma using inhaled steroid^36^. The authors hypothesized that inhaled steroid reduced peripheral blood T cell activation and Th2-type cytokine mRNA expression and acted as immunosuppressive and anti-proliferative agent^37^.

Our sub analyses showed that in subjects with AD, those with dyslipidemia showed greater risk of incident glaucoma than those without. A previous study also reported that AD in adolescence was associated with dyslipidemia (high LDL)^38,39^. Hyperlipidemia can induce and potentiate proinflammatory cytokines and a Th2 response to external antigens, which may be a link between the two diseases^40,41^. Also, in subjects with AR, current smokers showed greater risk of glaucoma than non-current smokers. Smoking is a well-established risk factor for both AR and glaucoma^42–45^. Smoking has been associated with worsening of AR symptoms and increased inflammatory biomarkers in AR, which may be the potential link^40,41^.

The present study has several strengths. This is the first study showing significant associations between the three allergic diseases and glaucoma risk in a large Nationwide longitudinal data. We were able to assess the temporal relationship between various allergic diseases and onset of glaucoma in various subgroups. In addition, we used physician-diagnosed rather than self-reported diseases for exposures, outcomes, and comorbidities, increasing the validity of the diagnosis. We were also able to adjust for comprehensive risk factors for glaucoma including lifestyle factors (smoking, drinking, and exercise) and systemic diseases (hypertension and dyslipidemia).

## Limitations

This study is subject to the following limitations. First, more detailed clinical information such as subtype or severity of diseases, which could lead to further understanding of the possible link between the two diseases, could not be assessed. Additionally, the possibility of detection bias cannot be ruled out. Patients with allergic diseases may visit hospitals more often, resulting in increased possibility of detecting glaucoma. In addition, our results were robust in all subgroups stratified by gender, lifestyle factors including smoking, drinking, and exercise, and systemic diseases including diabetes, hypertension, dyslipidemia, and history of steroid use.

## Conclusion

In conclusion, allergic diseases including AD, AR, and asthma were significantly associated with increased risk of glaucoma development in young adults. Our findings have clinical implications: because glaucoma is an irreversible progressive disease, screening for glaucoma in those with allergic diseases can be an effective strategy for early glaucoma diagnosis.

### Supplementary Information

Supplementary Table 1.

## Data Availability

The data that support the findings of this study are available from the Korea National Health Insurance Sharing Service (NHISS) Institutional Data Access Committee (https://nhiss.nhis.or.kr/bd/ay/bdaya001iv.do).
