# Supplementary material for: The risk of open angle glaucoma in young adults with allergic diseases: a Nationwide cohort study
Source: Sci Rep. 2024 May 10;14:10694. doi: 10.1038/s41598-024-57619-5 (PMC11082140; doi:10.1038/s41598-024-57619-5)
Supplement: Supplementary file 1 — Supplementary Table 1. [file 41598_2024_57619_MOESM1_ESM.docx]

Supplementary Table 1. Risk of primary open-angle glaucoma development in allergic diseases with at least one year lag

| 1 year Lag | N | POAG | Duration | IR (per 1,000) | Model 1 | Model 2 | Model 3 |
| --- | --- | --- | --- | --- | --- | --- | --- |
| **Atopic dermatitis, allergic rhinitis, or asthma** | | | | |  |  |  |
| No | 146789 | 4962 | 1072711.47 | 4.63 | 1 (ref.) | 1 (ref.) | 1 (ref.) |
| Yes | 23602 | 1176 | 163586.87 | 7.19 | 1.47 (1.38, 1.57) | 1.46 (1.37, 1.55) | 1.37 (1.28, 1.46) |
| **Atopic dermatitis** | | | | | | | |
| No | 169586 | 6085 | 1230900.57 | 4.94 | 1 (ref.) | 1 (ref.) | 1 (ref.) |
| Yes | 805 | 53 | 5397.77 | 9.82 | 2.04 (1.56, 2.67) | 2.03 (1.555, 2.66) | 1.78 (1.36, 2.34) |
| **Allergic rhinitis** | | | | | | | |
| No | 147922 | 5025 | 1080766.59 | 4.65 | 1 (ref.) | 1 (ref.) | 1 (ref.) |
| Yes | 22469 | 1113 | 155531.75 | 7.16 | 1.45 (1.36, 1.55) | 1.44 (1.35, 1.54) | 1.35 (1.26, 1.45) |
| **Asthma** |  |  |  |  |  |  |  |
| No | 168233 | 6026 | 1221349.62 | 4.93 | 1 (ref.) | 1 (ref.) | 1 (ref.) |
| Yes | 2158 | 112 | 14948.72 | 7.49 | 1.41 (1.17, 1.70) | 1.40 (1.16, 1.69) | 1.26 (1.04, 1.52) |
| Model 1 : Age, Sex | |  |  |  |  |  |  |
| Model 2 : Age, Sex, Income, hypertension, dyslipidemia, smoking, drinking, exercise, and body mass index | | | | | | |  |
| Model 3 : Age, Sex, Income, hypertension, dyslipidemia, smoking, drinking, exercise, body mass index, and steroid use | | | | | | | |

POAG: primary open-angle glaucoma
